# Fact‐finding survey by systematic review of active support groups accepting people with eating disorders in Japan

**DOI:** 10.1002/pcn5.183

**Published:** 2024-04-01

**Authors:** Sayo Hamatani, Yoshifumi Mizuno

**Affiliations:** ^1^ Research Center for Child Mental Development University of Fukui Fukui Japan; ^2^ Division of Developmental Higher Brain Functions, United Graduate School of Child Development University of Fukui Fukui Japan; ^3^ Department of Child and Adolescent Psychological Medicine University of Fukui Hospital Fukui Japan

## Abstract

This study systematically reviewed and listed support groups that are currently active in accepting people with eating disorders in Japan. The characteristics of the 50 support groups for eating disorders approved for publication are listed in Table 1 in Supplementary Materials.

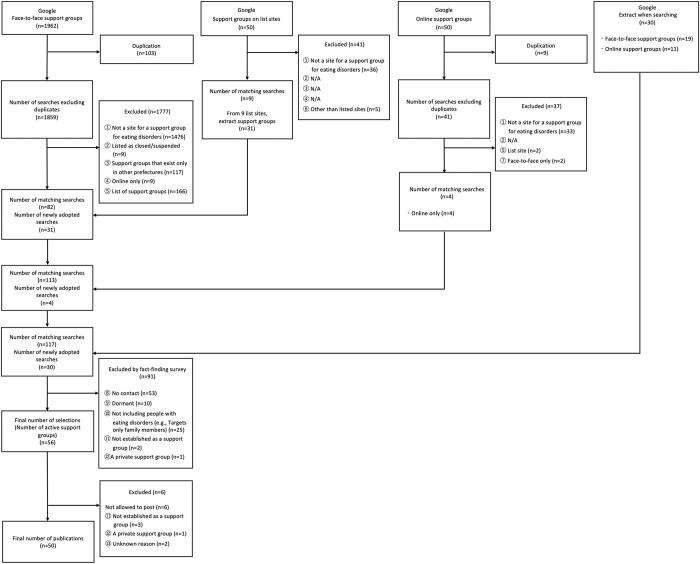

People with eating disorders can be extremely isolated due to shame and guilt,^1^ so they need a support system that enables them to receive support in their daily lives. Community care, such as self‐help groups and family associations, is a promising social framework for supporting people with eating disorders. Self‐help groups are groups in which people with the same problems gather to share mutual understanding and support,^2^ and it has been suggested that support groups may help increase individual involvement and motivation, reduce prejudice and isolation,^3^ and alleviate postprandial distress in young people with anorexia nervosa.^4^ However, to date, no study has systematically investigated support groups for Japanese people. Therefore, this study aimed to systematically review and list support groups that are currently active in accepting people with eating disorders in Japan.

A search for eating disorder support groups was conducted using the Google database as a systematic review method. The search terms were “eating disorder,” “self‐help group,” and the names of the 47 Japanese prefectures, and the top 50 results were selected if the number of results exceeded 50. Duplicates were excluded, and search results were selected based on the following exclusion criteria: (1) sites not belonging to eating disorder support groups; (2) support groups that are closed or inactive; (3) support groups not in the prefecture searched (support groups that exist only in other prefectures); (4) online‐only support groups; and (5) sites listing support groups. Subsequently, the same search was conducted to obtain a list of support groups. The search terms were “eating disorder self‐help groups, list,” and the results were extracted excluding those corresponding to (1), (2), (3), and (4) above, as well as (6) “support groups other than the listed sites.” Next, for online support groups, the search term was set to “eating disorder, self‐help group, online.” The results were extracted excluding those corresponding to (1), (2), and (5) above, as well as (7) support groups that only provided face‐to‐face support. In addition, support groups newly found in the search process were also extracted. Finally, among the 147 support groups extracted, those that could not be contacted by phone, e‐mail, or SNS after two attempts, and those who met criteria (9) to (12) during the fact‐finding survey were excluded, and active support groups were extracted (see Figure 1).

**Figure 1:**
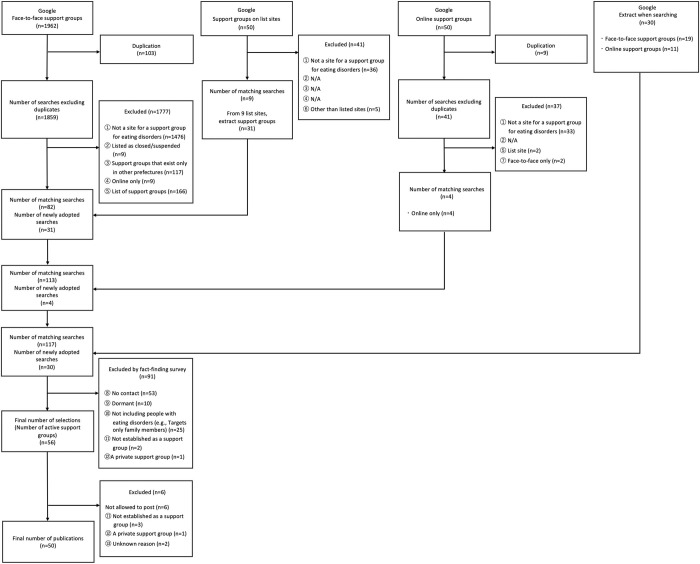
Flow diagram for the systematic review of active support groups in Japan.

Consequently, there were 56 active eating disorder support groups. The characteristics of the 50 support groups approved for publication are listed in Table S1 in Supplementary Materials. The number of active support groups per prefecture ranged from 0 to 4, with 19 (40%) regions having 0 support groups, accounting for approximately half of the total number of support groups. While there were several support groups in urban areas, such as Osaka and Kanagawa, the number of support groups was small in the Tohoku and Shikoku regions, indicating a sizeable regional maldistribution of support groups. The categories of support groups were self‐help groups in 43 cases (77%), family associations in four cases (7%), self‐help groups and family associations in two cases (4%) and others in seven cases (12%) (Figure S1). The meeting methods were as follows: 35 (62%) face‐to‐face, 16 (29%) face‐to‐face and online, and five (9%) online (Figure S2). Sixteen out of the 21 support groups meeting online (76%) started holding online events because of the COVID‐19 pandemic. Half (50%) of the support groups were held once a month, followed by every other week in 23% of the cases (Figure S3).

To our knowledge, there have been no systematic studies of active support groups for people with eating disorders in Japan. The list of active support groups attached to the appendix is a valuable tool for providing prompt access to a place of support. These lists allow clinicians and researchers to determine which referents are active in providing more party‐oriented support. The support recipients on these lists may also be potential collaborating agencies, and can provide an important basis for examining the impact and effectiveness of community care for people with eating disorders. The satisfaction of patients with eating disorders with social support is high,^5^ and there is a need to include other social support networks for them, in addition to medical care.^6^ Online support groups are also important, as holding meetings online allows participation from distant locations. However, this study had several limitations. Only Google was used as a search engine for this study. Since Google has a market share of over 80% in Japan,^7^ it is likely that most of the support groups were extracted; however, it is possible that a few groups could not be accessible using Google. Although active groups can be contacted immediately, the quality of support groups is unknown. Future studies are needed to evaluate the quality and effectiveness of support groups.

## FUNDING INFORMATION

This work was supported by Grants‐in‐Aid for Scientific Research from the Japan Society for the Promotion of Science (JSPS) (Grant Number 22H00985) and a Lotte Research Promotion Grant.

## CONFLICT OF INTEREST STATEMENT

The authors declare no conflicts of interest.

## ETHICS APPROVAL STATEMENT

This research did not require an ethical review as it did not fall under the ethical guidelines for life science and medical research involving human subjects.

## PATIENT CONSENT STATEMENT

All the support groups provided informed consent to participate in the study.

## CLINICAL TRIAL REGISTRATION

Not applicable.

## Supporting information

Supporting information.

Supporting information.

## Data Availability

A list of support groups for eating disorders is freely available in the supplemental materials.
