# Supplementary material for: Fact‐finding survey by systematic review of active support groups accepting people with eating disorders in Japan
Source: PCN Rep. 2024 Apr 1;3(2):e183. doi: 10.1002/pcn5.183 (PMC11114331; doi:10.1002/pcn5.183)
Supplement: Supplementary file 2 — Supporting information. [file PCN5-3-e183-s001.docx]

Supplementary Information

Figure S1 Categories of support groups

Figure S2 Method of meetings

Figure S3 Frequency of meetings
